# A New Perspective on Stroke Research: Unraveling the Role of Brain Oxygen Dynamics in Stroke Pathophysiology

**DOI:** 10.14336/AD.2024.0548

**Published:** 2025-08-02

**Authors:** Hongmei Zhou, Jialing Wang, Zhipeng Zhu, Li Hu, Erdan An, Jian Lu, Heng Zhao

**Affiliations:** ^1^Anesthesiology Department, The Second Hospital of Jiaxing, The Second Affiliated Hospital of Jiaxing University, Jiaxing, China.; ^2^Beijing Institute of Brain Disorders, Laboratory of Brain Disorders, Ministry of Science and Technology, Joint Innovation Center for Brain Disorders, Capital Medical University, Beijing, China

**Keywords:** Stroke, Green Enhanced Nano-Lantern, Hypoxic pockets, Oxygen dynamics

## Abstract

Stroke, a leading cause of death and disability, often results from ischemic events that cut off the brain blood flow, leading to neuron death. Despite treatment advancements, survivors frequently endure lasting impairments. A key focus is the ischemic penumbra, the area around the stroke that could potentially recover with prompt oxygenation; yet its monitoring is complex. Recent progress in bioluminescence-based oxygen sensing, particularly through the Green enhanced Nano-lantern (GeNL), offers unprecedented views of oxygen fluctuations in vivo. Utilized in awake mice, GeNL has uncovered hypoxic pockets within the cerebral cortex, revealing the brain's oxygen environment as a dynamic landscape influenced by physiological states and behaviors like locomotion and wakefulness. These findings illuminate the complexity of oxygen dynamics and suggest the potential impact of hypoxic pockets on ischemic injury and recovery, challenging existing paradigms and highlighting the importance of microenvironmental oxygen control in stroke resilience. This review examines the implications of these novel findings for stroke research, emphasizing the criticality of understanding pre-existing oxygen dynamics for addressing brain ischemia. The presence of hypoxic pockets in non-stroke conditions indicates a more intricate hypoxic scenario in ischemic brains, suggesting strategies to alleviate hypoxia could lead to more effective treatments and rehabilitation. By bridging gaps in our knowledge, especially concerning microenvironmental changes post-stroke, and leveraging new technologies like GeNL, we can pave the way for therapeutic innovations that significantly enhance outcomes for stroke survivors, promising a future where an understanding of cerebral oxygenation dynamics profoundly informs stroke therapy.

## 1. Introduction

Stroke is a leading cause of death and chronic disability, with ischemic stroke comprising most cases. This condition abruptly halts blood flow to parts of the brain, triggering harmful events that lead to neuron death [1, 2]. Despite progress in treatment and rehabilitation, the outcomes for many survivors are significantly impaired, highlighting the need for better understanding and treatments [3-5]. Ischemic stroke, making up about 87% of strokes, leads to complex reactions that worsen brain damage [6, 7]. The key to addressing ischemic stroke damage is the ischemic penumbra, tissue around the infarct that could be saved if blood flow is restored promptly, and oxygen supply is maintained [8-11]. However, tracking oxygen dynamics in these critical areas has been difficult, limiting our grasp of stroke pathology. Recent advancements in bioluminescence-based oxygen sensing, particularly using the genetically encoded bioluminescent oxygen indicator GeNL, have opened new pathways for directly observing dynamic oxygen processes in vivo [12]. The application of GeNL in awake, behaving mice has uncovered the presence of hypoxic pockets—localized areas of low oxygen—within the cerebral cortex, providing novel insights into the constantly fluctuating landscape of brain oxygenation [12]. These discoveries highlight the intricate complexity of oxygen dynamics in the brain under normal physiological conditions, and how these hypoxic pockets could significantly influence the process of ischemic injury and recovery. The identification of these hypoxic areas challenges previous understandings. It suggests that the microenvironmental control of oxygen levels may be crucial in mitigating ischemic damage and enhancing the brain's resilience to stroke.

Understanding the nuanced interplay between neuronal activity, blood flow, and oxygen availability is crucial for developing more targeted and effective treatments for ischemic stroke [13-17]. As we delve deeper into the significance of these findings, it becomes evident that the presence of hypoxic pockets in a non-stroke model provides a critical perspective on the cerebral microenvironment's vulnerability to ischemia [12]. This insight may not only illuminate the fundamental mechanisms potentially contributing to stroke-induced brain injury but also may open the door to novel therapeutic strategies aimed at mitigating ischemic damage and enhancing recovery. By identifying and understanding the factors that govern the formation and resolution of hypoxic pockets in healthy brain tissue, researchers can gain valuable clues on how to protect the brain against ischemic insult and promote recovery in stroke patients.

This review article explores the potential implications of these groundbreaking findings for stroke research, highlighting the importance of considering the pre-existing complexity of the brain's oxygen dynamics when addressing ischemic stroke-induced brain injury. The existence of hypoxic pockets in non-stroke conditions suggests that in the ischemic brain, the scenario of hypoxic stress and its impact on tissue viability and recovery is likely more complex. Therefore, strategies aimed at alleviating hypoxia and supporting oxygenation in the ischemic brain must account for this inherent complexity, potentially leading to more effective approaches for treatment and rehabilitation.

## 2. The critical gap in understanding the microenvironmental changes in the brain following an ischemic event, especially regarding oxygen dynamics

Building on the insights provided by recent advancements in bioluminescence-based oxygen sensing, particularly the GeNL technique, it is evident that traditional methods have not fully captured the complexity of brain oxygen dynamics. This sets the stage for addressing a crucial aspect of stroke pathology: the critical gap in understanding the microenvironmental changes in the brain following an ischemic event, especially regarding oxygen dynamics.

One of the critical gaps in our current understanding of stroke pathology lies in comprehending the microenvironmental changes occurring within the brain post-ischemia/reperfusion, especially with oxygen dynamics [18, 2]. While the gross mechanisms of ischemic stroke - including the abrupt reduction in cerebral blood flow and subsequent depletion of oxygen and nutrients - are well-documented [19, 20], the nuanced interplay of these factors at the microscale level remains less explored [21-23]. This lack of detailed insight is significant; it is within these microenvironments that the fate of neuronal tissue post-stroke is determined.

The concept of the ischemic penumbra highlights the importance of understanding these microenvironmental changes. The penumbra represents a threshold zone of injured yet viable brain tissue surrounding the infarct's core area [24, 25, 11]. The survival of this penumbral tissue is critically dependent on the restoration and regulation of oxygen supply. This balance is delicately maintained under normal physiological conditions but is severely disrupted during and after an ischemic stroke [26, 27]. After reperfusion post-stroke, there are dynamic changes in blood flow within the reperfusion region, leading to the complex and not fully understood "no-reflow" phenomenon [28]. This state, characterized by inadequate blood supply despite removing the occlusion, underscores the need for targeted strategies to enhance oxygen delivery and blood flow to the affected brain tissue, supporting recovery and minimizing further damage [29, 30]. The challenge has been to monitor and analyze these changes in real-time and with sufficient resolution to make meaningful observations that can inform treatment strategies.

## 3. Limitations in previous techniques and methods for evaluating brain oxygen level

Various techniques have been developed to monitor brain oxygen levels, each contributing uniquely to our understanding of cerebral oxygenation yet accompanied by specific limitations (Table 1). Traditional methods like Clark-type and oxygen microelectrodes provide direct measurements but are invasive and limited in spatial resolution, restricting their continuous application in vivo [31-33]. Laser-Doppler Flowmetry (LDF) and diffuse correlation spectroscopy offer insights into blood flow as a proxy for oxygenation. Yet, they lack the direct measurement of oxygen levels and have limited spatial resolution [34, 35]. While valuable, techniques such as phosphorescence quenching, often face challenges in real-time monitoring and can be invasive [36]. Advanced imaging modalities like Blood Oxygen Level Dependent (BOLD) MRI and Positron Emission Tomography (PET) bring the benefits of non-invasiveness and the ability to cover larger brain areas but rely on indirect measures of oxygenation, require expensive infrastructure, and offer limited temporal and spatial resolution [37, 38]. Near-Infrared Spectroscopy (NIRS) and its functional counterpart (fNIRS), along with Photoacoustic Imaging (PAI), provide useful insights into hemodynamic changes related to oxygenation. Yet, they are constrained by penetration depth and, in the case of PAI, remain largely experimental for brain applications [39, 40]. Electron Paramagnetic Resonance (EPR) Oximetry, although offering direct oxygen measurements, necessitates the implantation of probes, making it invasive [41].

**Table 1:** Overview of techniques for monitoring brain oxygen levels.

Techniques	Principle	Advantages	Limitations	References
Clark-type Electrodes	Direct measurement of oxygen through electrochemical reaction	High spatial and temporal resolution	Invasive, potential damage to tissue	[31-33]
LDF	Measures blood flow changes as a proxy for oxygenation	Non-invasive, real-time monitoring	Cannot directly measure oxygen levels, limited spatial resolution	[35]
Phosphorescence Quenching	Uses phosphorescent probes that change luminescence in response to oxygen levels	Can measure oxygen levels in tissue	Invasive (requires probe implantation for deep tissue)	[36]
BOLD MRI	Indirectly infers oxygenation through changes in blood flow and deoxyhemoglobin levels	Non-invasive, applicable to whole brain, functional imaging	Indirect measurement, susceptible to confounding factors	[37]
PET	Detects metabolic activity related to oxygen consumption using radioactive tracers	Quantitative, can image specific biochemical processes	Involves radioactive tracers, limited temporal resolution	[38]
NIRS	Optical method measuring changes in oxygenated and deoxygenated hemoglobin	Non-invasive, suitable for continuous monitoring	Limited penetration depth, semi-quantitative	[39]
PAI	Combines ultrasound and optical imaging to assess oxygenation based on hemoglobin properties	High spatial resolution, non-invasive	Experimental and complex setup, limited penetration depth	[40]
EPR Oximetry	Uses paramagnetic probes to measure oxygen levels through magnetic resonance changes	Direct measurement of tissue oxygenation	Requires implantation of probes, invasive	[41]

These traditional and advanced techniques collectively share overarching limitations, including indirect measurements of oxygen levels, invasiveness in certain cases, limited spatial and temporal resolutions, and the requirement for complex and expensive infrastructure. These constraints often prevent a comprehensive understanding of the oxygen distribution's dynamic and heterogeneous nature of oxygen distribution within the brain's microenvironments, particularly in real-time and in vivo contexts. In contrast, the GeNL technique emerges as a promising alternative, offering direct, non-invasive, real-time measurements of oxygen levels with high spatial resolution, thereby addressing many of the limitations inherent in previous methods and paving the way for novel insights into cerebral oxygenation dynamics [12].

## 4. The novel GeNL techniques to monitor oxygen changes in the brain

Navigating the complexities of cerebral oxygen dynamics, recent research utilizing the innovative GeNL technology has shed light on the ephemeral phenomenon known as hypoxic pockets within the mouse cerebral cortex. This exploration delves into their formation, characteristics, and implications for understanding the brain's oxygen landscape. The study unveils a nuanced vista of cerebral microenvironmental variability by closely examining the onset, resolution, and spatial distribution of these transient oxygen-deprived zones, alongside meticulous validation against potential imaging artifacts. The findings challenge previous perceptions of uniform oxygen distribution and underscore the intricate interplay between vascular architecture, microcirculation, and neuronal activity in sustaining cerebral health and functionality [12].

### 4.1 The evolution and impact of nano-lantern technology in biomedical research

The Nano-lantern technology, innovated by Takeharu Nagai and his team, offers a solution to the limitations of traditional fluorescence imaging, such as the need for external light and issues with phototoxicity [42]. By fusing a Renilla luciferase variant with a fluorescent protein, the Nano-lantern emits a strong fluorescent signal activated by a luciferase substrate like furimazine, through bioluminescence resonance energy transfer (BRET) [43]. Since its creation in 2012, the technology has expanded to include a variety of colors, enhancing its application range from optogenetics to biosensing [44-47, 42]. Introducing diacetyl coelenterazine-h advanced the technology by preventing autooxidation and background fluorescence, thus enabling longer imaging durations [48].

**Figure 1.**
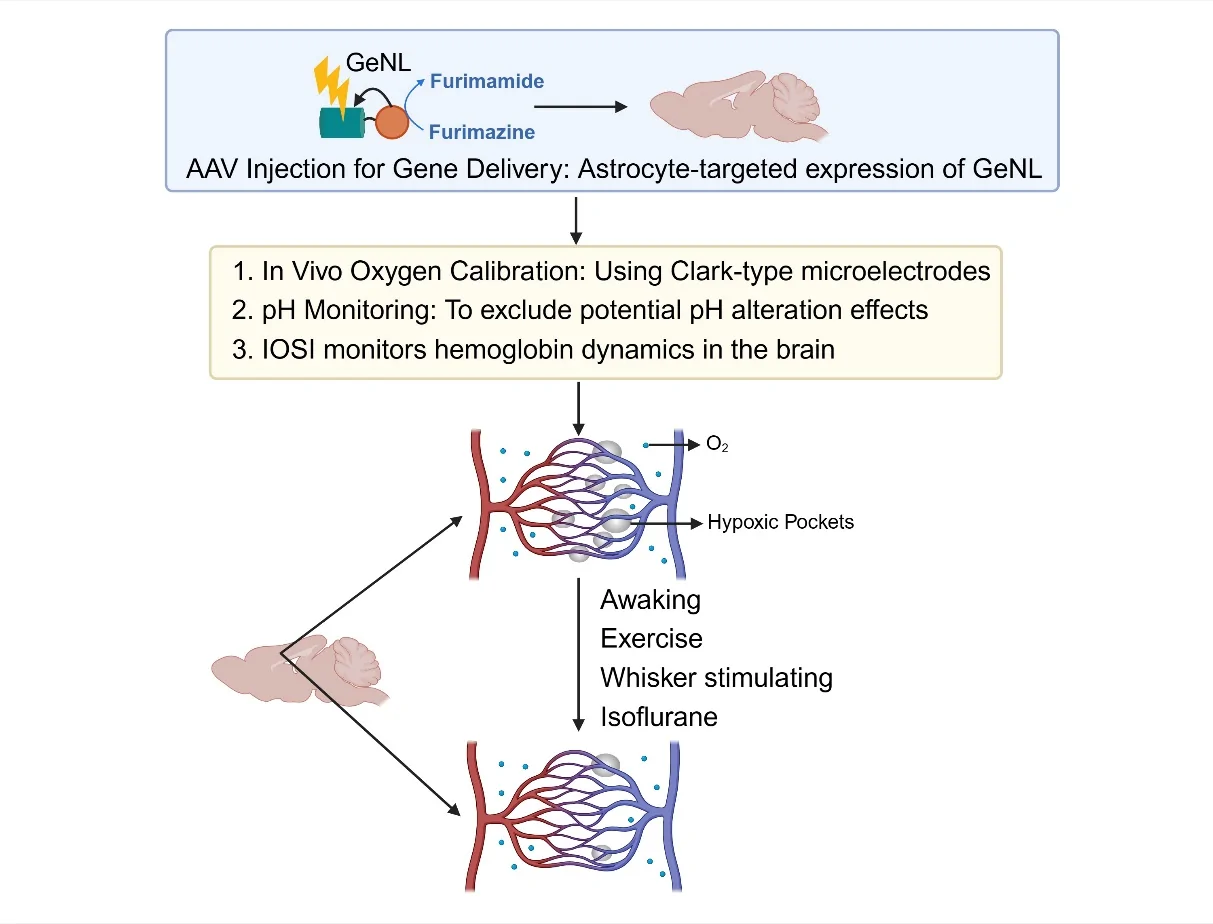
**Schematic representation of the methodology for monitoring cerebral oxygenation and hemodynamic responses using GeNL**. The process begins with AAV-mediated gene delivery for the astrocyte-targeted expression of the GeNL followed by the administration of furimazine that leads to a bioluminescent reaction in the presence of oxygen, producing furimamide. The enzymatic reaction produces a bioluminescent signal captured as bioluminescence intensity (BLI). The intensity correlates with the local partial oxygen tension (PO2), allowing for the visualization of oxygen dynamics. This process enables the detection of "hypoxic pockets," regions with transiently reduced PO2. The figure demonstrates the method's application in awake mice, highlighting its utility in monitoring cerebral oxygenation changes and understanding the physiological relevance of oxygen distribution in the brain. Key investigative techniques include: (1) In vivo oxygen calibration using Clark-type microelectrodes to measure partial oxygen tension (PO2); (2) pH monitoring to ensure that the observed bioluminescence changes are not due to pH fluctuations; and (3) intrinsic optical signal imaging (IOSI) to assess hemoglobin dynamics reflecting blood flow in the brain. The lower section demonstrates how changes in physiological states (awakening, exercise, whisker stimulation, and isoflurane administration) influence oxygenation, denoted by the appearance of hypoxic pockets, indicated by the blue dots. The network of vessels is depicted with arterioles in red and venules in blue, symbolizing the distribution of oxygen-rich and oxygen-poor blood, respectively, and illustrating the areas where hypoxic pockets are likely to form in response to various stimuli. This integrative approach enables the visualization and quantification of localized oxygen fluctuations and the understanding of their behavioral and physiological modifiers.

Recent developments have led to enhanced Nano-lanterns (eNLs) that use the bright luciferase NanoLuc and its substrate furimazine, boosting the brightness significantly and enabling multicolor imaging and even single-molecule visualization (Fig. 1). GeNL, a variant within the eNL spectrum, exemplifies the potential of this technology, which utilizes bioluminescence resonance energy transfer (BRET) for the real-time measurement of oxygen levels in biological tissues [12]. At its core, GeNL comprises two main components: NanoLuc Luciferase, derived from deep-sea shrimp and engineered for enhanced light output, and mNeongreen Fluorescent Protein, which is genetically fused to NanoLuc [45, 49]. The interaction begins when NanoLuc catalyzes the oxidation of the substrate furimazine in the presence of oxygen, leading to light production. This light then transfers energy to mNeongreen through BRET, causing it to fluoresce brightly. This bioluminescence intensity correlates with oxygen levels, enabling the visualization of oxygen distribution within tissues [43, 49].

The GeNL technique monitors oxygen dynamics within the cerebral cortex, specifically focusing on hypoxic pockets [12]. This technique begins with the expression of GeNL in astrocytes, glial cells that extensively infiltrate the brain's neuropil, allowing for widespread oxygen monitoring. The genetic encoding of GeNL is achieved using adeno-associated viruses (AAVs) that carry the gene constructs for NanoLuc luciferase and mNeongreen fluorescent protein. These AAVs are delivered into the brain through precise stereotactic injections, ensuring targeted expression within astrocytes.

Once the AAVs infect the astrocytes, the genetic material integrates into the host cells, leading to the expression of the bioluminescent components [12]. Further, furimazine, a substrate for NanoLuc luciferase, is administered systemically. When furimazine is metabolized by the NanoLuc enzyme, it produces a bioluminescent signal, the intensity of which is directly related to the local oxygen concentration due to the oxygen-dependent nature of the oxidation process.

The bioluminescent signal is captured using bioluminescence imaging (BLI), which employs specialized cameras and imaging systems to detect low-light signals [12]. These systems quantify the light intensity, correlating it with the local oxygen levels in real-time. This real-time monitoring allows researchers to observe dynamic changes in oxygen levels within the brain, providing immediate feedback on fluctuations such as those seen in hypoxic pockets.

The procedure begins with the preparation of mice, including creating a cranial window for direct visualization of the brain's cortical surface and injecting AAVs carrying the GeNL construct [12]. After allowing time for expression, furimazine is administered, and the animals are placed under an imaging system equipped with a sensitive CCD camera. The emitted bioluminescent light is captured and translated into data reflecting oxygen levels. This data is then processed and analyzed to quantify the bioluminescent signal intensity and monitor changes over time, providing insights into the distribution and dynamics of oxygen within the brain's microenvironment. This method enables the visualization and quantification of oxygen levels in living, behaving animals, offering valuable information on the role of oxygen in brain function and pathology.

### 4.2 The discovery and validation of hypoxic pockets

The comprehensive study delves into the dynamic nature of oxygen tension within the cerebral cortex, elucidating the spontaneous emergence of localized transient reductions in oxygen levels, termed "hypoxic pockets." Utilizing the innovative GeNL technology, researchers have offered a window into the cerebral microenvironment's complexity under resting conditions, challenging prior assumptions of uniform oxygen distribution across the brain.

Through continuous imaging, the study identified hypoxic pockets within the mouse cerebral cortex as localized, transient reductions in oxygen levels with sharply defined borders and rapid onset and resolution. These phenomena were recorded throughout 20-minute sessions, with an average of 200 occurrences in anesthetized mice. They affected roughly 2.43% of the visual field and exhibited a nearly circular shape. Notably, these hypoxic pockets often reemerged in the same cortical areas, indicating a predisposition of certain regions to transient hypoxic events.

The research further compared these occurrences to induced hypoxia conditions (10% inhaled O2), finding comparable reductions in partial pressure of oxygen (PO2) levels, thus affirming the hypoxic character of these pockets. Efforts to exclude hemoglobin absorption artifacts confirmed that the mNeonGreen fluorescence of GeNL, though susceptible to hemoglobin absorption, did not display patterns mirroring hypoxic pockets, eliminating potential biases in imaging.

Analysis revealed that hypoxic pockets formed nearer to venules than arterioles, suggesting a nuanced vascular influence on the emergence of these oxygen-deprived zones. The hemodynamic basis for hypoxic pocket formation was explored, revealing that changes in microcirculation, particularly around areas of low total hemoglobin concentration ([HbT]), paralleled the dynamics of hypoxic pockets as seen in intrinsic optical spectroscopy imaging (IOSI) [12]. This connection between capillary blood flow and tissue oxygenation lends significant insight into cerebral hypoxia mechanisms, highlighting the critical role of vascular architecture and microcirculatory patterns in developing and localizing of hypoxic pockets within the brain tissue. This detailed examination of hypoxic pockets, their hemodynamic origins, and the relationship with surrounding vascular structures offers profound insights into the cerebral microenvironment. It opens new avenues for exploring therapeutic strategies aimed at mitigating ischemic damage and enhancing recovery in stroke patients, emphasizing the necessity of maintaining optimal microcirculatory conditions to safeguard brain health.

### 4.3 Activity-induced modulation of hypoxic pockets: insights into cerebral oxygenation mechanisms

The study explores the effects of vasodilation and capillary stalling on the formation and dynamics of hypoxic pockets within the brain's cortex, contributing significant insights into cerebral oxygenation and its regulatory mechanisms. Vasodilation, induced either by hypercapnia (increased CO2 levels) or isoflurane anesthesia, significantly reduces the number and size of hypoxic pockets. Hypercapnia, in particular, led to a 41% reduction in the number of hypoxic pockets and a 53% decrease in the area they covered, with the PO2 within these pockets being less reduced during hypercapnia and staying low after that [12]. This suggests that by increasing tissue oxygenation, vasodilation might counterbalance the formation of hypoxic areas, potentially through increased O2 diffusion. Interestingly, hyperoxia (elevated oxygen levels in inhaled air) had a less pronounced effect on the reduction of hypoxic pockets than vasodilation, indicating that vasodilation more effectively controls the prevalence of these pockets than mere blood oxygenation.

Furthermore, the study investigates the phenomenon of capillary stalling—where circulating leukocytes halt capillary blood flow—by introducing microspheres to occlude capillaries [12]. This intervention led to a change in the dynamics of hypoxic pockets, with a decrease in their number but an increase in the total area affected, suggesting that hypoxic pockets near occluded areas may merge. This interaction between microsphere-induced blockages and local oxygen dynamics underscores the critical role of capillary circulation in maintaining tissue oxygenation and preventing hypoxic conditions.

The study reveals that wakefulness and locomotion significantly affect the dynamics of hypoxic pockets within the mouse cerebral cortex. In awake mice, particularly those allowed to engage in locomotion (e.g., running on a polystyrene ball), the presence of hypoxic pockets—localized, transient reductions in oxygen tension—was markedly reduced compared to those in ketamine-xylazine (KX) anesthetized mice [12]. Specifically, the number of hypoxic pockets in awake, behaving mice decreased by 17% and further dropped by 35% in those actively running, indicating a substantial reduction in the occurrence of these pockets with increased activity levels.

Interestingly, while the number of hypoxic pockets decreased with wakefulness and locomotion, the surface area of these pockets increased by 41% in awake mice, suggesting a more extensive but less frequent oxygen deficit under these conditions [12]. Additionally, the duration of hypoxic pockets was shorter in awake mice by approximately 8 seconds. Yet, the amplitude of the pockets remained consistent, pointing towards a dynamic oxygen environment influenced by the state of consciousness and physical activity.

In awake behaving mice, stimulating the whisker barrel cortex through contralateral whisker stimulation led to an increase in BLI, indicating a rise in local PO2 [12]. This finding illustrates that BLI can detect sensory-induced changes in cortical oxygen levels, with a notable difference in the amplitude and timing of the BLI response between awake and anesthetized states, highlighting the dampening effect of anesthesia on tissue PO2 and functional hyperemia dynamics.

The study further demonstrated that the reduction in hypoxic pockets during active locomotion was not simply due to a generalized increase in blood flow. Instead, these pockets’ highly structured spatial characteristics were preserved, underscoring a complex relationship between neuronal activity, blood flow, and oxygenation in the brain. This is supported by the finding that sensory stimulation, like whisker stimulation in anesthetized mice, also led to a decrease in hypoxic pockets, highlighting the role of functional hyperemia—increased blood flow to areas of the brain engaged in activity—in mitigating the formation of these pockets [12].

In summary, the research underscores the dynamic nature of cerebral oxygenation, which is significantly influenced by the behavioral state of the animal. Wakefulness and particularly locomotion decrease the frequency and duration of hypoxic pockets, potentially reflecting enhanced neuronal activity and blood flow in these states. These findings offer new insights into the mechanisms underlying cerebral oxygenation and its regulation by physiological behaviors, suggesting potential strategies for minimizing hypoxic conditions within the brain.

### 4.4 Potential limitations of the GeNL technique

Despite its significant advancements in real-time, noninvasive monitoring of brain oxygen dynamics, the GeNL technique presents certain limitations and challenges [12]. One major limitation is its inability to provide absolute quantification of oxygen levels. GeNL primarily detects relative changes, which can be insufficient when precise oxygen measurements are necessary for understanding specific pathophysiological conditions. The technique excels at monitoring short-term oxygen fluctuations but may be less effective for long-term baseline comparisons across different experimental conditions or groups.

Another limitation is the potential for imaging artifacts, such as those caused by hemoglobin absorption. However, studies have validated that these artifacts do not mimic the patterns of hypoxic pockets, ensuring the reliability of the observed oxygen dynamics. The accuracy of GeNL in reflecting cerebral oxygen levels has been further reinforced through calibration with Clark-type microelectrodes.

The choice of astrocyte-specific expression for GeNL might overlook the impacts of other cellular or molecular structures on oxygen diffusion [12]. While it is presumed that oxygen diffuses freely across the neuropil, this assumption requires careful consideration when interpreting results.

Applying GeNL necessitates a sophisticated imaging setup to capture bioluminescence signals, including precise gene delivery and substrate administration. The technique’s complexity demands rigorous control and calibration procedures to maintain accuracy and reliability during real-time monitoring [12].

Given the limitations of the GeNL technique, traditional methods can provide essential complementary data. Despite being invasive, Clark-type electrodes offer direct and absolute measurements of oxygen levels with high spatial and temporal resolution, making them invaluable for baseline comparisons and precise quantification [50]. Techniques such as BOLD MRI and PET provide non-invasive imaging capabilities over larger brain areas, offering a broader context for understanding cerebral oxygenation, albeit indirectly [51, 52]. Additionally, although invasive and technically demanding, phosphorescence quenching and EPR oximetry, although invasive and technically demanding, deliver direct tissue oxygenation measurements [53, 54]. These methods can complement GeNL by quantifying and validating the observed oxygen dynamics, ensuring a comprehensive understanding of brain oxygenation.

In essence, the GeNL technique represents a significant advancement in monitoring brain oxygen dynamics, providing valuable insights into the formation and modulation of hypoxic pockets. Despite its limitations, such as the inability to provide absolute oxygen measurements and the potential for imaging artifacts, the application of GeNL has illuminated critical aspects of cerebral oxygenation. Future improvements and complementary approaches could further enhance its utility, paving the way for a better understanding and treatment of conditions like ischemic stroke.

## 5. Implications of hypoxic pockets for stroke research enhanced understanding of ischemic pathophysiology

This novel study of GeNL about hypoxic pockets provides crucial clues for understanding what happens during a stroke. It sheds new light on the cerebral microenvironment's nuanced response to ischemia, significantly enriching our understanding of oxygen deprivation and the complexities of reperfusion injury. By revealing the existence and behavior of hypoxic pockets within the awake mouse cortex, the research illustrates how localized fluctuations in oxygen availability can impact neuronal health and function [12]. These insights emphasize the importance of microenvironmental conditions in determining the outcome of ischemic events, moving beyond the traditional focus on blood flow blockages to consider how oxygen is distributed and utilized at the tissue level. This enhanced understanding of ischemic pathophysiology could lead to more precise diagnostic and prognostic tools, allowing clinicians to better predict the extent of stroke damage and recovery potential.

As demonstrated by the study, the ability to monitor and manipulate oxygen levels in real-time, provides a powerful platform for modeling ischemic stroke conditions [12, 55]. By artificially inducing hypoxic pockets or alleviating them through interventions such as induced locomotion, researchers can mimic the effects of ischemic stroke and reperfusion in a controlled manner. This approach offers invaluable insights into the localized and systemic effects of stroke, including the role of oxygen deprivation in neuronal injury and the potential for recovery through reoxygenation. Furthermore, it allows for exploring how behavioral and physiological changes—akin to rehabilitation exercises in humans—can influence the course of recovery, providing a more holistic view of stroke recovery mechanisms.

Understanding the dynamics of hypoxic pockets and their susceptibility to modulation opens up new avenues for therapeutic intervention [12]. Specifically, it highlights the potential for targeting the microcirculatory and oxygenation processes within the brain to mitigate ischemic damage. Therapies that enhance tissue oxygenation, improve capillary blood flow, or prevent the formation of hypoxic pockets could significantly reduce the severity of ischemic injury. Additionally, interventions aimed at boosting the brain's intrinsic mechanisms for dispersing or compensating for hypoxic pockets—such as promoting physical activity or optimizing wakefulness—may offer non-invasive strategies for enhancing brain resilience to ischemia [56, 57]. Identifying the molecular and cellular pathways that regulate hypoxic pocket dynamics could lead to developing novel pharmacological agents designed to protect the brain from ischemic damage and accelerate recovery.

The role of brain oxygen dynamics in stroke outcomes becomes particularly significant with aging [58]. As the brain ages, its ability to maintain adequate oxygenation and respond to ischemic events diminishes. This is due to vascular changes such as reduced vessel elasticity, arteriosclerosis, and impaired blood flow autoregulation, all of which exacerbate hypoxic conditions during a stroke [58]. These changes may increase the incidence of hypoxic pockets, worsening neuronal injury and delaying recovery. Moreover, the aged brain's reduced capacity for neuroplasticity and repair makes it more vulnerable to hypoxia and ischemia [59]. Understanding these age-related challenges can help develop targeted therapeutic strategies to mitigate the impact of hypoxic pockets and improve stroke outcomes. By incorporating age-specific considerations into research and treatment protocols, we can enhance intervention efficacy and provide better care for older stroke patients. This underscores the importance of personalized approaches in stroke therapy for an aging population [60].

The role of brain oxygen dynamics in stroke outcomes is particularly significant when considered in the context of aging. As the brain ages, its ability to maintain adequate oxygenation and respond to ischemic events diminishes due to vascular changes like reduced vessel elasticity, arteriosclerosis, and impaired blood flow autoregulation, exacerbating hypoxic conditions during a stroke [58]. These changes may increase the incidence of hypoxic pockets, worsening neuronal injury and delaying recovery. Additionally, the aged brain's reduced capacity for neuroplasticity and repair makes it more vulnerable to hypoxia and ischemia [59]. Understanding these age-related challenges can help develop targeted therapeutic strategies to mitigate the impact of hypoxic pockets and improve stroke outcomes. By incorporating age-specific considerations into research and treatment protocols, we can enhance intervention efficacy and provide better care for older stroke patients, emphasizing the importance of personalized approaches in stroke therapy for an aging population [60].

In summary, the findings from this study provide a foundation for a new era in stroke research, one that recognizes the critical role of tissue oxygenation and microenvironmental integrity in the pathophysiology of ischemic stroke. By focusing on the dynamics of hypoxic pockets, researchers and clinicians can explore innovative strategies to prevent, treat, and rehabilitate ischemic brain injury, potentially transforming care for stroke patients worldwide.

## 6. Challenges and future directions

Translating findings from mouse models to human stroke therapy involves several challenges and strategies to overcome these obstacles. One primary challenge is the anatomical and physiological differences between mice and humans. Mice have smaller brain sizes, different blood vessel distribution, and distinct metabolic rates, all of which can influence the onset, progression, and recovery from ischemic events. These differences can influence the onset, progression, and recovery from ischemic events [61-63]. Additionally, the human brain's complexity, with its intricate network of neurons and vasculature, presents a more complicated environment for understanding and treating ischemic stroke [64]. Therefore, while mouse models provide critical insights into the fundamental mechanisms underlying stroke, the leap to human applicability requires cautious interpretation and further validation.

To effectively bridge the gap between animal models and human stroke therapy, a comprehensive approach is necessary, incorporating cross-species studies, the application of advanced imaging technologies, and interdisciplinary collaboration. Developing studies in larger animal models that more closely resemble human brain structure and stroke effects can provide a smoother transition of mouse model findings into clinical practices [65, 62]. Additionally, leveraging sophisticated imaging and monitoring technologies in human research could unveil deeper insights into capillary flow, neuronal activity, and oxygen dynamics, crucial for identifying therapeutic opportunities and assessing treatment impact in real-time [66, 67].

The complexity of ischemic stroke, underscored by the nuanced interplay of brain oxygen dynamics, calls for collaborative efforts that cross traditional disciplinary boundaries [68-70]. By bringing together neuroscientists, clinicians, bioengineers, and data scientists, novel tools and methodologies can be developed, facilitating the translation of laboratory discoveries into effective clinical solutions for stroke recovery. Moreover, exploring how therapeutic interventions can modulate capillary flow and oxygenation post-stroke, including pharmacological agents and lifestyle modifications like exercise, is vital for advancing treatment options [71, 72].

Understanding hypoxic pockets and brain oxygen dynamics paves the way for potential therapeutic interventions such as remote conditioning and ischemic postconditioning, which can significantly improve stroke outcomes [5, 73]. Remote conditioning, by inducing brief ischemic episodes in remote tissues, could enhance brain resilience to hypoxia by optimizing oxygen delivery and reducing hypoxic stress [73]. Ischemic postconditioning, involving controlled interruptions of blood flow post-reperfusion, can minimize reperfusion injury by stabilizing oxygen levels and preventing oxidative damage [74, 5]. Additionally, therapies improving microcirculation, targeted neuroprotective agents, and behavioral interventions like exercise could further mitigate hypoxic pockets, collectively offering innovative strategies for stroke treatment and recovery [75, 76].

By addressing these areas, the stroke research community can build upon existing knowledge, moving closer to developing treatments that significantly improve stroke survivors' lives. Adhering to these multidimensional recommendations will accelerate advancements in stroke care, offering new hope and possibilities for recovery and rehabilitation.

## 7. Conclusion

The findings on hypoxic pockets and brain oxygen dynamics using the GeNL technique significantly impact stroke research and therapy. These insights improve our understanding of ischemic microenvironmental changes and enable more targeted interventions, such as remote conditioning and ischemic postconditioning, to enhance oxygen delivery and modulate reperfusion, thereby reducing ischemic damage and aiding recovery. Complementing GeNL with traditional methods ensures precise quantification and validation, providing a comprehensive understanding of brain oxygenation.

The future of stroke research will focus on the interplay between microcirculatory dynamics and neuronal health. Advanced imaging and real-time monitoring will foster innovative, personalized therapies, improving stroke outcomes. Continued exploration of brain oxygen dynamics promises to unlock new pathways for neuroprotection and recovery, transforming stroke care.
